# Genotypic virulence profiles and associations in *Salmonella* isolated from meat samples in wet markets and abattoirs of Metro Manila, Philippines

**DOI:** 10.1186/s12866-022-02697-6

**Published:** 2022-12-06

**Authors:** Rance Derrick N. Pavon, Paolo D. G. Mendoza, Camille Andrea R. Flores, Alyzza Marie B. Calayag, Windell L. Rivera

**Affiliations:** Pathogen-Host-Environment Interactions Research Laboratory, Institute of Biology, College of Science, University of the Philippines Diliman, Quezon City, 1101 Philippines

**Keywords:** Association, Pathogenicity islands, Prevalence, *Salmonella*, Virulence genes

## Abstract

**Background:**

*Salmonella* are pathogenic foodborne bacteria with complex pathogenicity from numerous virulence genes housed in *Salmonella* pathogenicity islands (SPIs), plasmids, and other gene cassettes. However, *Salmonella* virulence gene distributions and mechanisms remain unestablished. In the Philippines, studies mainly report *Salmonella* incidences and antimicrobial resistance, but little to none on virulence profiles, their associations to animal sources, collection sites and *Salmonella* serogroups. Hence, a total of 799 *Salmonella* isolates, previously obtained from pig, cow, and chicken meat samples in wet markets and abattoirs (wet markets: 124 chicken, 151 cow, and 352 pig meat isolates; abattoirs: 172 pig tonsil and jejunum isolates) in Metro Manila, Philippines, were revived and confirmed as *Salmonella* through *invA* gene polymerase chain reaction (PCR). Isolates were then screened for eight virulence genes, namely *avrA*, *hilA*, *sseC*, *mgtC*, *spi4R*, *pipB*, *spvC* and *spvR*, by optimized multiplex PCR and significant pair associations between virulence genes were determined through Fisher’s exact test. Gene frequency patterns were also determined. *Salmonella* serogroups in addition to animal sources and location types were also used to predict virulence genes prevalence using binary logistic regression.

**Results:**

High frequencies (64 to 98%) of SPI virulence genes were detected among 799 *Salmonella* isolates namely *mgtC*, *pipB*, *avrA*, *hilA*, *spi4R* and *sseC,* from most to least. However, only one isolate was positive for plasmid-borne virulence genes, *spvC* and *spvR*. Diversity in virulence genes across *Salmonella* serogroups for 587 *Salmonella* isolates (O:3 = 250, O:4 = 133, O:6,7 = 99, O:8 = 93, O:9 = 12) was also demonstrated through statistical predictions, particularly for *avrA*, *hilA*, *sseC*, and *mgtC*. *mgtC*, the most frequent virulence gene, was predicted by serogroup O:9, while *sseC*, the least frequent, was predicted by serogroup O:4 and chicken animal source. The highest virulence gene pattern involved SPIs 1-5 genes which suggests the wide distribution and high pathogenic potential of *Salmonella*. Statistical analyses showed five virulence gene pair associations, namely *avrA* and *hilA*, *avrA* and *spi4R*, *hilA* and *spi4R*, *sseC* and *spi4R*, and *mgtC* and *pipB*. The animal sources predicted the presence of virulence genes, *sseC* and *pipB*, whereas location type for *hilA* and *spi4R*, suggesting that these factors may contribute to the type and pathogenicity of *Salmonella* present.

**Conclusion:**

The high prevalence of virulence genes among *Salmonella* in the study suggests the high pathogenic potential of *Salmonella* from abattoirs and wet markets of Metro Manila, Philippines which poses food safety and public health concerns and threatens the Philippine food animal industry. Statistical associations between virulence genes and prediction analyses across *Salmonella* serogroups and external factors such as animal source and location type and presence of virulence genes suggest the diversity of *Salmonella* virulence and illustrate determining factors to *Salmonella* pathogenicity. This study recommends relevant agencies in the Philippines to improve standards in food animal industries and increase efforts in monitoring of foodborne pathogens.

## Background

*Salmonella* are Gram-negative, rod-shaped, facultative anaerobic, and motile pathogenic bacteria within the *Enterobacteriaceae* family [1] that commonly reside in food animals such as livestock and poultry. They are one of the leading causes of food-borne illnesses [2]. Extensive diseases, whether gastroenteritis to systemically disseminated infections, are brought about by numerous antigenic variations among more than 2600 serovars [3–5]. Despite this, the genus *Salmonella* is divided only into *S. enterica* and *S. bongori*, with the former further divided into six subspecies [6]. However, subspecies I (*S. enterica* subsp. *enterica*) is the only one often associated with diseases among mammals. This includes Enteritidis, Typhimurium, and Typhi, which commonly cause infections [7]. *Salmonella* classification depends on the antigenic characterization of O (somatic), H (flagellar), and Vi (capsular) antigens and is traditionally performed through the White-Kauffmann-Le Minor scheme for serological testing [8]. The O-antigen corresponds to the O-polysaccharide found in the outermost layer of Gram-negative cells. It varies in structure and sugar composition, thus providing discrimination of *Salmonella* serogroups, such as B (O:4), C1 (O:6,7), C2-3 (O:8), D (O:9), and E (O:3,10) [9–12]. Meanwhile, H antigens allow differentiation of serovars and primarily have two types; phase 1 and phase 2 flagellin proteins, encoded by *fliC* and *fliB* genes, respectively [13–15]. In the Philippines, several studies in genotyping *Salmonella* using multiplex polymerase chain reaction (PCR) of O, H1, and H2 associated genes have been conducted in *Salmonella* from abattoirs and wet markets in Metro Manila [16–19].

*Salmonella* pathogenicity islands (SPIs) contain a plethora of virulence genes encoding for type III secretion systems (T3SS), transcriptional regulators, transporters, host immune interference proteins, and effectors that mediate invasion within host intestinal cells [20]. With currently 23 SPIs, containing numerous virulence factors and may have different distributions and genetic stabilities across *Salmonella* serovars [21, 22], *Salmonella* pathogenesis remains complex and largely unknown. The most studied SPI is SPI1, which is 40 kb in size and contains virulence genes such as *inv*, *avr*, *hil*, *spa*, *sip*, among others, and encodes for the T3SS responsible for contact-dependent transport of effector protein complexes into host cells hence contributing to invasion, pathogenesis, and host inflammatory pathways [23]. SPI2, also a well-studied SPI and 40 kb in size, encodes for another T3SS distinct from SPI1 and is activated intracellularly required for *Salmonella* replication [22, 23]. It contains effectors such as *sse*, *sif*, *sop*, *srf*, *ssp*, among others, that affect *Salmonella*-containing vacuole positioning, host cytoskeleton, and immune signaling [24]. Meanwhile, SPI3, although less studied and only 17 kb in size, has been involved with intramacrophage survival and primarily contains *mgt*, *mis* and *mar* genes [23]. SPI4 is 27 kb in size and has largely unknown functions although has been shown to encode a type 1 secretion system (T1SS) and mediates in adhesion [23, 25]. It harbors *sii* genes involved with immune modulation and bacterial internalization [26]. Lastly, SPI5 is only 7 kb in size with roles in enteropathogenicity encoding genes such as *pip* which have been associated with lipid raft accumulation and intramacrophage survival [22, 23, 27]. Meanwhile, plasmid-borne virulence genes in *Salmonella*, particularly *spv* genes, have only been found in a small number of subspecies I *Salmonella* serovars such as Choleraesuis, Dublin, Typhimurium and Enteritidis, among others, with variable sizes and contributes to increased pathogenicity, *Salmonella* replication in animals and systemic infections in humans [28]. Factors such as the amount of viable *Salmonella* ingested, *Salmonella* serovar and pathogenicity, and host status can also influence the clinical outcome [29]. Virulence genes were previously shown to be differentially expressed among *S. enterica* serovars and strains. Invasive forms of Enteritidis and Typhimurium, for example, exhibited repression of SPI1 and SPI4 virulence genes. In contrast, less invasive serovars Infantis and Hadar exhibited upregulation during intramacrophage infection experiments [30]. In contrast, a comparison of invasive and non-invasive phenotypes within a serovar Typhimurium strain from phase-variation, showed that SPI1 virulence genes, such as those encoding flagellins and bacterioferritin, were upregulated in invasive phenotypes [31]. Besides incidence, serogroup and serovar data [12, 16–19] and in silico serotyping using *invA* virulence gene [32], there is little to no information about the prevalence of virulence-associated genes in *Salmonella* in the Philippines with only *spvC* being detected from wet markets of Metro Manila [16]. Hence, this study detects virulence genes through multiplex PCR of *Salmonella* that were previously isolated and serogrouped in earlier studies from various retail meat of pig, cow, or chicken origins in wet markets, and pig tonsils and jejuna in abattoirs of Metro Manila. This study also determines statistical associations among virulence genes and predictions of their prevalence by *Salmonella* serogroups and external factors.

## Methodology

### Revival of *Salmonella* isolates

A total of 799 *Salmonella* isolates were previously collected from 2013 to 2016 by the Pathogen-Host-Environment Interactions Research Laboratory, Institute of Biology, College of Science, University of the Philippines Diliman from various meat samples obtained from wet markets and slaughtered pig tonsil and jejunum samples from abattoirs of Metro Manila, Philippines [12, 18, 19]. Of the 799 isolates, only 587 were previously subjected to molecular serogrouping, while the remaining 212 isolates were either not previously subjected to serogrouping or possessed putative identities [12, 19]. For wet market location type, various meat samples resulted in a total of 672 isolates wherein 151 isolates were from cows, 124 from chickens and 352 from pigs. Various meat samples included different meat products in retail wet markets such as different parts and raw or processed meats of the three animal sources. For abattoir location type, there were a total of 172 isolates, all from tonsils and jejuna of slaughtered pigs. In the case of *Salmonella* serogroups, 250 belong to the O:3 serogroup, 133 to O:4, 99 to O:6,7, 93 to O:8, and 12 to O:9. Culture-based isolation of *Salmonella* from these studies followed standard protocols [16, 17, 19]. Additionally, confirmed isolates stored in glycerol stocks at − 20 °C were subjected to a revival process for this study based on protocols from previous studies [12, 19] with some modifications. Briefly, 100-μL glycerol stock culture was transferred to 900-μL trypticase soy broth (TSB) (BD Diagnostics System, NJ, USA) and incubated at 37 °C for 18-24 h. Then, a loopful of TSB culture was streaked onto xylose lysine deoxycholate (XLD) agar (BD Diagnostics System, NJ, USA) plates and incubated at 37 °C for 18-24 h. Typical *Salmonella* colonies were then sub-cultured and purified on nutrient agar (NA) (BD Diagnostics System, NJ, USA) for DNA extraction and molecular confirmation through *invA* gene detection.

### DNA extraction

DNA extraction was conducted using the boil-lysis method [16, 17, 19]. Two to three colonies of *Salmonella* grown on NA for 18-24 h at 37 °C were suspended in 50-μL 1X Tris-EDTA (TE) buffer and heated at 100 °C for 10 min. After cooling to room temperature, suspensions were then centrifuged at 2656 x g for 5 min. The supernatant, which contains the DNA was then transferred to a new sterile microcentrifuge tube and stored at − 20 °C for subsequent assays.

### Molecular confirmation of *Salmonella*

DNA extracts in TE buffer were subjected to confirmatory PCR for *Salmonella* by amplifying and detecting the *invA* gene based on protocols from previous studies [16, 17, 32]. Each PCR reaction was 12.5 μL in volume, which consisted of 6.25 μL 2× GoTaq Green Master Mix (Promega, WI, USA), 4.25 μL nuclease-free water, 0.5 μL each of 10 μM forward and reverse primers for *invA* gene, and 1 μL DNA template. Descriptions, primer sequences, amplification conditions, amplicon size, and corresponding references for *invA* gene can be found in Table 1.

**Table 1:** Descriptions, primer sequences, amplicon sizes, amplification conditions and references for *Salmonella* virulence genes

Target Virulence Gene	Description	*Salmonella* Pathogenicity Island	Primers	Sequences	Amplicon Size	Amplification Conditions						Reference
						ID	D	A	E	Cycles	FE	
*invA*	invasion protein	1	*invA*^F^	5′-ACAGTGCTCGTTTACGACCTGAAT-3’	244 bp	95 °C 2 min	95 °C 30 s	60 °C 30 s	72 °C 30 s	30x	72 °C 5 min	[33]
			*invA*^R^	5′-AGACGACTGGTACTGATCTAT-3’								
*avrA*	putative inner membrane protein	1	*avrA*^F^	5′-GTTATGGGACGGAACGACATCGG-3’	385 bp	94 °C 4 min	94 °C 1 min	58 °C 2 min	72 °C 2 min	35x	72 °C 5 min	[34]
			*avrA*^R^	5′-ATTCTGCTTCCCGCCGCC-3’								
*sseC*	secretion system effector	2	*sseC*^F^	5′-TATGGTAGGTGCAGGGGAAG-3’	121 bp							[35]
			*sseC*^R^	5′-CTCATTCGCCATAGCCATTT-3’								
*mgtC*	Mg^2+^ transport protein	3	*mgtC*^F^	5′-TGACTATCCAATGCTCCAGTGAAT-3’	655 bp							[36]
			*mgtC*^R^	5′-ATTTACTGGCCGCTATGCTGTTG-3’								
*pipB*	pathogenicity island encoded protein from SPI5	5	*pipB*^F^	5′-TAATGTGCCACATACAGGTAACGC-3’	789 bp							[37]
			*pipB*^R^	5′-TTCTGGAGGATGTCAACGGGTG-3’								
*hilA*	invasion genes transcription activator/regulator	1	*hilA*^F^	5’CTGCCGCAGTGTTAAGGATA-3’	497 bp	95 °C 3 min	95 °C 30 s	50 °C 30 s	72 °C 30 s	35x	72 °C 5 min	[34]
			*hilA*^R^	5′-CTGTCGCCTTAATCGCATCGT-3’								
*spvR*	*Salmonella* plasmid virulence for regulation of *spv* operon	Plasmid	*spvR*^F^	5′-ATGGATTTCATTAATAAAAAATTA-3’	894 bp							[38]
			*spvR*^R^	5′-TCAGAAGGTGGACTGTTTCAGTTT-3’								
*spvC*	*Salmonella* plasmid virulence: hydrophilic protein	Plasmid	*spvC*^F^	5′-ACTCCTTGCACAACCAAATGCGGA-3’	571 bp	95 °C 3 min	95 °C 30 s	50 °C 30 s	72 °C 30 s	35x	72 °C 5 min	[33]
			*spvC*^R^	5′-TGTCTTCTGCATTTCGCCACATCA-3’								
*spi4R*	type I secretion system protein	4	*spi4R*^F^	5′-GATATTTATCAGTCTATAACAGC-3’	1269 bp	94 °C 4 min	94 °C 1 min	58 °C 1 min	72 °C 2 min	35x	72 °C 5 min	[39]
			*spi4R*^R^	5′-ATTCTCATCCAGATTTGATGTTG-3’								

^F^ Forward and ^R^ Reverse primers, *ID* Initial denaturation, *D* Denaturation, *A* Annealing, *E* Extension, *FE* Final extension

### Multiplex PCR optimization and detection of virulence genes

Multiplex and singleplex assays to detect eight virulence genes, namely *avrA*, *sseC*, *mgtC*, *pipB*, *spi4R*, *hilA*, *spvC*, and *spvR*, representing SPIs 1–5 and plasmid-borne genes, were optimized by temperature gradient PCR and used to screen *invA* confirmed *Salmonella*. Each multiplex PCR reaction was 12.5 μL in volume, which consisted of 6.25 μL 5X MyTaq HS Red Mix (Bioline, London, UK), 0.25 μL each of 10 μM forward and reverse primers, and 2 μL DNA template in TE buffer while variable amounts of nuclease-free water depending on the number of primer sets used to make up for the 12.5 μL volume. Each singleplex PCR reaction was also 12.5 μL in volume and followed the same composition as *invA* gene PCR, except for *spi4R*, which required 20 μM primer concentrations. *avrA*, *sseC*, *mgtC*, and *pipB* genes were optimized for multiplex PCR. Meanwhile, *hilA* and *spvR* genes were also optimized for multiplex PCR with the same conditions as with *spvC*, which was conducted in singleplex PCR. This is due to lack of amplification if all three genes (*hilA*, *spvR* and *spvC*) were included in the multiplex reaction. Amplification of the *spi4R* gene was optimized in singleplex PCR. Similar to *invA* gene, descriptions, primer sequences, amplification conditions, amplicon sizes, and corresponding references for virulence genes investigated in this study can be found in Table 1. Through Kwik-Stik™ (Microbiologics), *S. enterica* subsp. *enterica* ATCC (American Type Culture Collection) serovars Typhimurium (ATCC 14028) and Enteritidis (ATCC 13076) were used as positive controls for *invA*, *avrA*, *sseC*, *mgtC*, *pipB*, and *spi4R*, and Choleraesuis (7001) for *hilA*, *spvC*, and *spvR*. Negative controls used include *Escherichia coli* (ATCC 35218) and *Klebsiella pneumoniae* (ATCC 7881) also through Kwik-Stik™.

### Gel electrophoresis and visualization

All PCR amplicons were analyzed in 2% agarose gels (Vivantis, Malaysia) in 1x Tris-Acetate-EDTA (TAE) with 10,000x SYBR® Safe DNA Gel Stain (ThermoFisher Scientific, MA, USA). 5 μL PCR products were loaded in each well with KAPA 10kbp Universal Ladder (Kapa Biosystems, MA, USA) as the molecular weight marker. Electrophoresis was conducted at 280 V for 45 min using the CBS Scientific gel electrophoresis system (ThermoFisher Scientific, MA, USA) containing 1× TAE solutionas the running buffer. Gels were then viewed using the Vilber Lourmat gel documentation system (Vilber, France).

### Data analysis

Frequencies and patterns of virulence genes across *Salmonella* isolates and serogroups were determined and visualized with Excel Office 365 (Microsoft) and R version 4.0.5 in RStudio (R Foundation). UpSetR 1.4.0 was used to generate intersection plots for virulence gene patterns in RStudio [40, 41]. All statistical analyses were conducted in SPSS version 1.0.0.1447 (IBM). Significant associations in pairs among virulence genes across *Salmonella* isolates were determined using Fisher’s exact test, a descriptive statistical analysis in place of Chi-Square under cross tabulations. Binary logistic regression was used to determine whether *Salmonella* serogroup (O:3, O:4, O:6,7, O:8, or O:9), animal source (pig, cow, or chicken), or location type (wet market or abattoir) as independent variables can predict the presence of virulence genes as dependent variables, thereby assessing their contributions to *Salmonella* virulence [42]. For *Salmonella* serogroup, O:3 was used as a reference category, the pig for animal source, and wet market for location type. Odds ratios and *p*-values were determined to signify predictive effects on virulence genes prevalence. The statistical significance of all analyses was based on *p*-value less than 0.05. Analyses excluded *invA* virulence gene since it was used as a marker for *Salmonella* spp. confirmation.

## Results

### Prevalence of virulence genes across *Salmonella* isolates

A total of 799 *Salmonella* isolates were successfully revived using culture-based methods and were positive for *invA* virulence gene, confirming their *Salmonella* identity. All virulence genes located in SPIs 1–5 showed more than 60% prevalence (Tables 2 and 3). Among them, the most frequently detected gene was *mgtC* (98.62%), followed by *pipB* (97.37%), *avrA* (88.24%), *hilA* (71.21%), *spi4R* (65.71%), and *sseC* (64.71%). In contrast, plasmid virulence genes *spvC* and *spvR* were only detected in one isolate (0.13%).

**Table 2:** Prevalence of virulence genes in *Salmonella* based on animal source

		Virulence Genes							
		SPI1		SPI2	SPI3	SPI4	SPI5	Plasmid	
	No. of isolates	***avrA***	***hilA***	***sseC***	***mgtC***	***spi4R***	***pipB***	***spvC***	***spvR***
**Total**	799	705 (88.24%)	569 (71.21%)	517 (64.71%)	788 (98.62%)	525 (65.71%)	778 (97.37%)	1 (0.13%)	1 (0.13%)
**Pig**	524	472 (90.08%)	376 (71.76%)	322(61.45%)	514 (98.09%)	322 (61.45%	517 (98.66%)	1 (0.19%)	1 (0.19%)
**Cow**	151	125 (82.78%)	106 (70.2%)	103(68.21%)	150 (99.34%)	116 (76.82%)	142 (94.04%)	0 (0%)	0 (0%)
**Chicken**	124	108 (87.1%)	87 (70.16%)	92(74.19%)	124 (100%)	87 (70.16%)	119 (96.97%)	0 (0%)	0 (0%)

**Table 3:** Prevalence of virulence genes in *Salmonella* based on location type

		Virulence Genes							
		SPI1		SPI2	SPI3	SPI4	SPI5	Plasmid	
	No. of isolates	***avrA***	***hilA***	***sseC***	***mgtC***	***spi4R***	***pipB***	***spvC***	***spvR***
**Total**	799	705 (88.24%)	569 (71.21%)	517 (64.71%)	788 (98.62%)	525 (65.71%)	778 (97.37%)	1 (0.13%)	1 (0.13%)
**Wet market**	627	545 (86.92%)	430 (68.58%)	418 (66.67%)	619 (98.72%)	444 (70.81%)	608 (96.97%)	0 (0%)	0 (0%)
**Abattoir**	172	160 (93.02%)	139 (80.81%)	99 (57.56%)	169 (98.26%)	81 (47.09%)	170 (98.84%)	1 (0.58%)	1 (0.58%)

### Prevalence of virulence genes among *Salmonella* serogroups

**Fig. 1:**
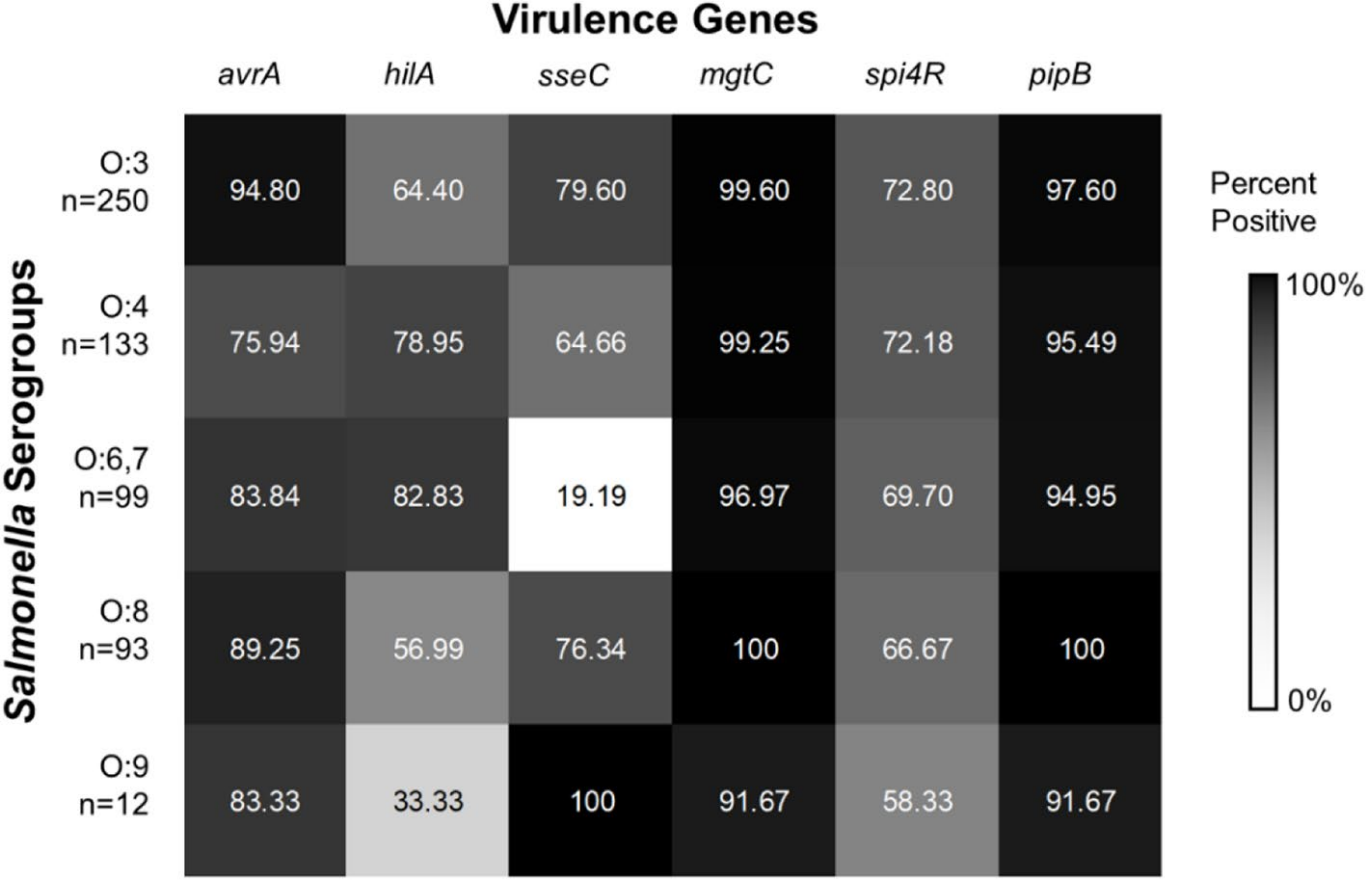
Frequencies of six (6) virulence genes across *Salmonella* serogroups, excluding *spvC* and *spvR* which are both not found across 587 serogrouped *Salmonella* isolates

Considering the 587 *Salmonella* isolates that were previously subjected to molecular serogrouping [12, 19], serogroup-based variations in virulence gene frequencies were observed. While *mgtC* and *pipB* showed a high prevalence (> 90%) across all serogroups, other genes, such as *avrA, spi4R*, *sseC*, and *hilA* showed drastic variations across *Salmonella* serogroups (Fig. 1). For *avrA*, the highest frequency was observed in O:3 (94.80%) while the lowest in O:4 (75.94%), and for *spi4R*, the highest was also in O:3 (72.80%) while the lowest in O:9 (58.33%). Interestingly, while other *Salmonella* serogroups showed more than 60% prevalence of *sseC*, O:6,7 only showed a 19.19% detection rate. Similarly, O:9 only had a 33.33% prevalence of *hilA* as compared to more than 50% in other serogroups.

Binary logistic regression showed significant predictions (*p* < 0.05) for the presence of some virulence genes among *Salmonella* serogroups (Table 4). *avrA* and *sseC* were predicted by serogroups O:4 and O:6,7, while *hilA* was predicted by serogroups O:4, O:6,7 and O:9, and *mgtC* was predicted by serogroup O:9 all relative to O:3 reference group, while *spi4R* and *pipB* had no significant predictions (*p* > 0.05). Odds ratio values revealed that serogroups O:4 and O:6,7 were less likely to carry *avrA* (*p*-values = < 0.001, 0.001; odds ratios = 0.173, 0.285) and *sseC* (*p*-values = 0.002, < 0.001; odds ratios = 0.469, 0.061), but more likely to have *hilA* (*p*-values = 0.004, < 0.001; odds ratios = 2.073, 2.666) relative to O:3. Serogroup O:9, alternatively, was shown to be less likely positive for *hilA* (*p*-value = 0.040, odds ratio = 0.276) and *mgtC* (*p*-value = 0.031, odds ratio = 0.044) relative to O:3.

**Table 4:** Binary logistic regression on whether *Salmonella* serogroups predict virulence genes prevalence

	***Salmonella*** Serogroups							
	O:4^**a**^		O:6,7^**a**^		O:8^**a**^		O:9^**a**^	
Gene	*p*-value	odds ratio	*p*-value	odds ratio	*p*-value	odds ratio	*p*-value	odds ratio
***avrA***	< 0.001*	**0.173**	0.001*	**0.285**	0.073	0.455	0.117	0.274
***hilA***	0.004*	**2.073**	< 0.001*	**2.666**	0.209	0.732	0.040*	**0.276**
***sseC***	0.002*	**0.469**	< 0.001*	**0.061**	0.513	0.827	0.999	4.140 × 10^8^
***mgtC***	0.665	0.530	0.077	0.129	0.997	6.487 × 10^6^	0.031*	**0.044**
***spi4R***	0.897	0.969	0.561	0.859	0.266	0.747	0.282	0.523
***pipB***	0.266	0.52	0.212	0.462	0.997	3.972 × 10^7^	0.244	0.27

*Significant predictor to virulence gene presence if *p* < 0.05, ^a^Relative to O:3 serogroup (reference category was chosen based on their larger sample size)

### Patterns and associations of virulence genes

**Fig. 2:**
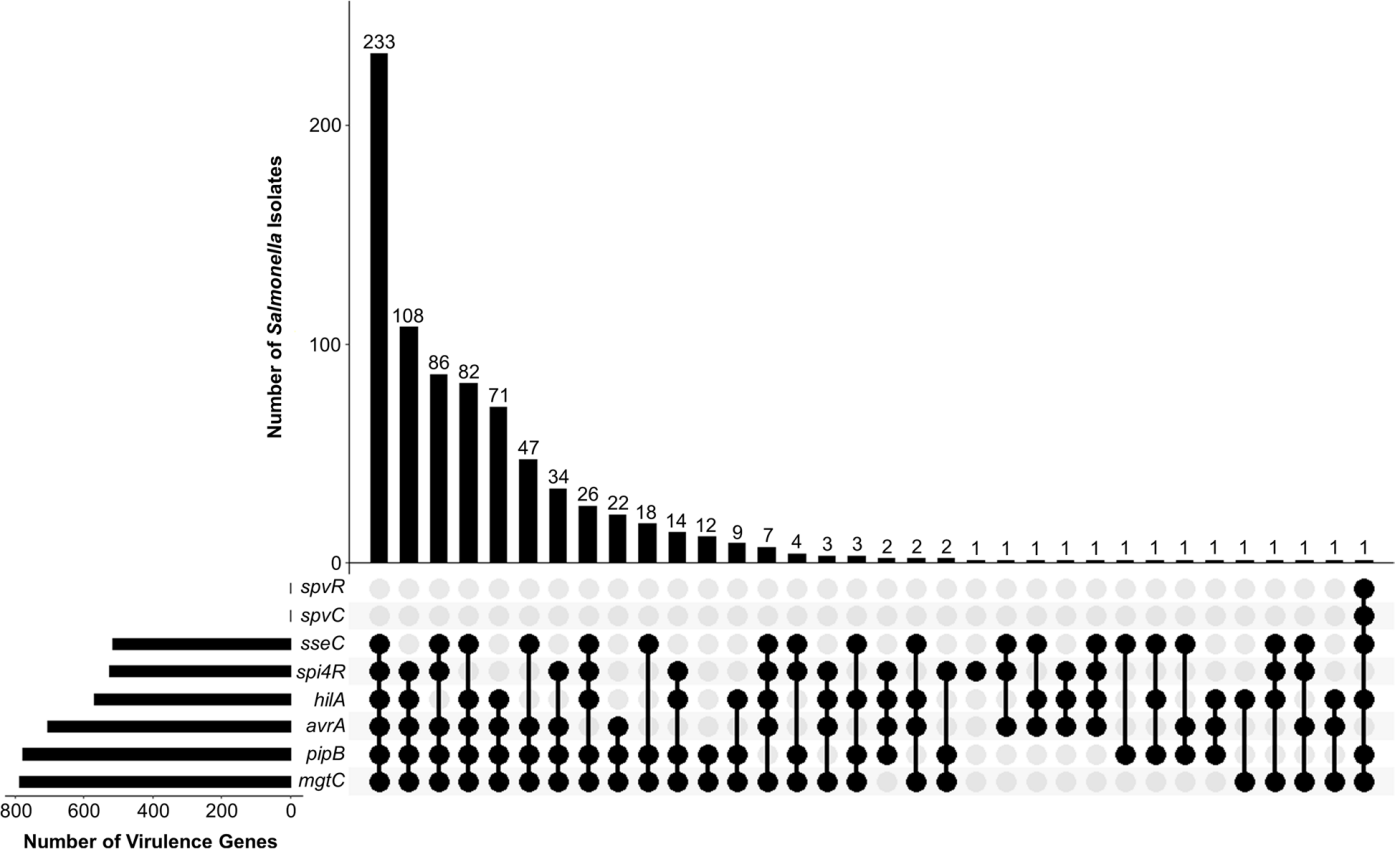
Virulence gene patterns across 799 *Salmonella* isolates

Among all 799 *Salmonella* isolates, the most frequent (29.16%) gene combination involved six genes which were *avrA*, *hilA*, *sseC, mgtC*, *pipB*, *spi4R*, and *sseC* (Fig. 2), which encompasses SPIs 1–5. This is immediately followed by three combination patterns containing five virulence genes wherein 13.52% with *spi4R*, *hilA*, *avrA*, *pipB*, and *mgtC*, 10.76% with *sseC*, *spi4R*, *avrA*, *pipB*, and *mgtC*, and 10.26% with *sseC*, *spi4R*, *avrA*, *pipB*, and *mgtC*. The virulence gene patterns among *Salmonella* isolates showing five or more gene combinations at higher frequencies suggest their overall high pathogenic potential. While the single isolate with *spvC* and *spvR* also contained *hilA*, *sseC*, *mgtC*, and *pipB* genes. Statistical analysis using Fisher’s exact test between virulence gene pairs uncovered five significant associations (*p* < 0.05), namely, *avrA* and *hilA*, *avrA* and *spi4R*, *hilA* and *spi4R*, *sseC* and *spi4R*, and *mgtC* and *pipB* (Table 5). *spvC* and *spvR* were excluded in all statistical analyses as they only occurred in one isolate.

**Table 5:** Associations between virulence gene pairs across *Salmonella* isolates

Virulence Genes Associations	Fisher’s Exact Test (two-sided ***p***-values)
***avrA*and*hilA***	0.011*
*avrA* and *sseC*	0.206
*avrA* and *mgtC*	0.129
***avrA*and*spi4R***	0.002*
*avrA* and *pipB*	0.728
*hilA* and *sseC*	0.102
*hilA* and *mgtC*	0.523
***hilA*and*spi4R***	< 0.001*
*hilA* and *pipB*	0.220
*sseC* and *mgtC*	0.531
***sseC*and*spi4R***	0.002*
*sseC* and *pipB*	1.000
*mgtC* and *spi4R*	0.525
***mgtC*and*pipB***	< 0.001*
*spi4R* and *pipB*	0.360

*Significant association if *p* < 0.05

### External factors affecting virulence gene prevalence

Variations in virulence gene prevalence were observed across animal sources and location types (Tables 2 and 3). *mgtC* and *pipB* genes showed a high prevalence (> 90%). *sseC* and *spi4R* prevalence were lower among pigs than other animal sources and lower in abattoirs than wet markets. *hilA* were found less frequently in wet markets than in abattoirs but had similar frequencies across different animal sources. *spvC* and *spvR* were detected only in one isolate; which was obtained from a pig host source in an abattoir location type.

Using binary logistic regression, animal sources and location types in this study could significantly (*p* < 0.05) predict some virulence gene occurrences. *sseC* and *pipB* were predicted by the animal source chicken and cow respectively, relative to pigs. In contrast, *hilA* and *spi4R* were predicted by location type abattoir, relative to the wet market. Meanwhile, *avrA* and *mgtC* showed no significant predictions (*p* > 0.05) (Table 6). Odds ratio values for animal sources relative to pigs suggest that *Salmonella* from chickens were more likely to carry *sseC* (*p*-value = 0.029; odds ratio = 1.663), while cows were less likely to carry *pipB* (*p*-value = 0.009; odds ratio = 0.227). For location type, *Salmonella* from abattoirs were more likely to carry *hilA* (*p*-value = 0.001; odds ratio = 2.044) but less likely to carry *spi4R* (*p*-value = < 0.001, odds ratio = 0.41) relative to wet markets.

**Table 6:** Contribution of animal source relative to pig and location type relative to market on virulence genes prevalence using binary logistic regression

	Animal Source				Location Type	
	Cow^**a**^		Chicken^**a**^		Abattoir^**b**^	
Gene	*p*-value	odds ratio	*p*-value	odds ratio	*p*-value	odds ratio
***avrA***	0.077	0.616	0.647	0.865	0.118	1.709
***hilA***	0.527	1.143	0.561	1.141	0.001*	**2.044**
***sseC***	0.296	1.241	0.029*	**1.663**	0.201	0.785
***mgtC***	0.300	3.043	0.996	3.278 × 10^7^	0.848	1.143
***spi4R***	0.059	1.526	0.726	1.083	< 0.001*	**0.410**
***pipB***	0.009*	**0.227**	0.095	0.343	0.810	1.225

*Significant predictor to virulence gene presence if *p* < 0.05, ^a^Relative to pig animal source, ^b^Relative to wet market location type. Reference categories were chosen based on their larger sample sizes

## Discussion

In this study, nine *Salmonella* virulence genes were investigated, with most genes except for *spvC* and *spvR* showing more than 61.45% prevalence. The occurrence of *mgtC* and *pipB* is similar to the findings of Fazl et al. [35] or at higher frequencies than some studies. Joaquim et al. [43] reported a similar prevalence of *mgtC* (98.31%) and *avrA* (93.22%) among *Salmonella* from slaughtered pigs, and from intensive or backyard farms with also low incidence of *spvC* (5.08%). Meanwhile, less than 50% of *Salmonella* from feces, organs, and transrectal swabs of healthy swine in farms of Tuscany, Central Italy, carried *mgtC* and *pipB* [44]. Similarly, a study on poultry-associated *Salmonella* reported less than 60% prevalence of these genes, but a higher prevalence (52%) of *spv* (e.g., R, C, B) genes than the current study [45]. *mgtC*, found within SPI3, is activated under low Mg^2+^ concentration, low pH, or in the presence of antimicrobial peptides, such as within host macrophages, which enables the transport of Mg^2+^ crucial for growth and survivability [46, 47]. *mgtC* complementation experiments have also been reported to restore wild-type phenotypes of SPI3 mutant *Salmonella*, suggesting the significance of *mgtC* within SPI3 [48]. *pipB*, found in SPI5 and translocated by T3SS [28], also promotes intramacrophage survival [37] and is involved in the accumulation of lipid rafts [23]. *avrA*, the third most detected virulence gene among *Salmonella* isolates in this study and within SPI1, has been demonstrated to mediate intracellular survival [49] through reduction of Beclin-1 protein, suppression of autophagy [50], and activation of STAT3 pathway involved in carcinogenesis [51]. The *hilA*, also in SPI1, is a central transcriptional regulator for other genes within the SPI [52]. In contrast to this study, others showed a 100% *avrA* or *hilA* gene occurrence among *Salmonella* from poultry, such as chicken and pigeons [34, 53, 54] and tested on specific serovars, such as Enteritidis and Typhimurium. Comparatively, 80% of *Salmonella* isolated from retail beefs in Malaysia and South Africa also possessed *hilA* which interestingly showed variations among serovar Agona and Enteritidis while none had *spvC*, albeit a low number of isolates [55, 56]. Whole-genome sequencing of serovars, such as Infantis strain Sal147 revealed numerous deletions of SPI1 genes such as *avrA*, *hilA*, and even *invA* [57]. Similarly, *Salmonella* from chickens in Egypt showed the absence of *avrA* in serovars Molade, Bargny, Inganda, and Infantis [58]. *Salmonella* serovar Heidelberg in a study of poultry abattoirs in Brazil showed a comparable prevalence of *hilA* (66.6%) and higher frequencies of *avrA* (98.4%) [59]. These studies collectively suggest serovar-dependent variations in virulence gene frequencies, which may explain the frequencies of *avrA* and *hilA* in this study. The role of SPI4, containing *sii* genes, is still unknown [23] but encodes a type 1 secretion system (T1SS) [22], mediates adhesion [26], or may also be involved in intramacrophage survival and toxin production [39]. Alternately, *sseC* is required to facilitate protein translocation through insertion into phagosome membranes [60]. The lower occurrence of *sseC* in this study can be compared with *Salmonella* from diarrheic children in hospitals which showed only 1.7% occurrence [61]. However, this may be due to differences in hosts sources which have been shown to cause variations in *Salmonella* serovars present and subsequent virulence gene prevalence. Other studies involving poultry and humans showed a 100% occurrence of *sseC* [35]. This study’s low frequency of *spv* genes may be due to associations with specific serovars [59]. On the other hand, Chaudhary et al. [62] showed that while 81% of *Salmonella* isolates (Typhimurium and Enteritidis) had *spvR*, none had *spvC*. An earlier study in Metro Manila, Philippines, showed different occurrence rates of *spvC* among *Salmonella* serogroups isolated from wet markets with the highest frequencies among O:7 and O:4 serogroups [12]. In contrast, another study on *Salmonella* isolates from retail beef showed that all eight different serovars were negative for *spvC* [61].

Most of the isolates possess five or more virulence genes, which reflect other studies that illustrate SPIs 1–5 as prevalent among serovars, whereas other SPIs are variably distributed across *Salmonella* [22]. However, Rychlik et al. [63] evaluated the pathogenicity of mutant *Salmonella* Enteritidis among chickens and found that only SPIs 1–2 were crucial for systemic infection while SPIs 3–5 individually had little effect on colonization capacity. A core genome among invasive *Salmonella*, involving SPIs 1–5, 9, 13, and 14 as well as other genes, was determined by a study using the microarray technique but phenotypically showed noninvasive strains having superior intracellular replication over the invasive strains [64]. This study also identified significant virulence gene co-occurrences. The co-occurrence of *spvC* and *spvR* was not tested in this study due to their low occurrence, which may be attributed to both being within an accessory virulence locus with *spvR* as the transcriptional regulator among nontyphoidal *Salmonella*, while their presence may still enhance pathogenicity to extraintestinal levels [65]. Virulence gene associations between *hilA* with *avrA* and *spi4R* may be explained by its role as a major regulatory gene, while *avrA* and *spi4R* may be due to other mechanisms between interactions of SPI1 and SPI4 virulence factors [66]. The *siiA* gene, within SPI4 and encoding an effector involved in T1SS-dependent adhesion of *Salmonella* [67], has been reported in vitro and in vivo as a direct target of *hilA* binding and differential regulation, thereby affecting SPI4 expression and the invasion process [68]. In contrast, *sseC* functions as a translocon component chaperoned by SscA within the SPI2 T3SS [69], *sii* genes in SPI4 are designated to form the T1SS to secrete the SiiE effector protein [70]. However, crosstalk mechanisms remain unestablished. The association between *mgtC* and *pipB* may be due to their similar involvement in *Salmonella* such as their induction in intramacrophage environments [37]. Similarly, transcriptome analysis by RNA-seq of *Salmonella* Typhimurium under intramacrophage conditions showed upregulation of the *mgtCBR* operon and SPI5 genes, such as *pipB* [71]. Nonetheless, the associations between genotypic variabilities and pathogenicity in *Salmonella* remain unclear and require further studies [72]. Virulence gene expressions which contribute to *Salmonella* pathogenicity are affected by numerous factors such as signals, nutrient limitation and other stresses including possible relationships with antimicrobial resistance [73–75].

In contrast with the current findings on external factors, Mthembu et al. [42] showed that the animal source and sample type did not significantly predict the prevalence of *Salmonella* virulence genes in their study of small-scale commercial farms but were instead predicted by location. Animal health conditions may also be a factor for the observed virulence gene predictions in this study. Skyberg et al. [76] compared virulence gene profiles of *Salmonella* from healthy and clinically ill birds and showed that some virulence genes, such as *sopB* were more frequently detected in the latter while others, such as *lpfC* and *sifA* were more frequently detected in the former, suggesting diverse roles in pathogenicity. The *sopE* was not found among prevalent serovars isolated from animals in Senegal but was present in all serovars isolated from diarrheic children and animals in Gambia [77], suggesting an interplay of animal source, health condition, and location in *Salmonella* serovar and virulence genes prevalence. Virulence gene patterns may also vary across a more specific location (e.g., market stalls), wherein a unique pattern, for example, was observed in chicken meat isolate from a given stall, while other patterns were more common in all stalls in a study of *Salmonella* recovered from wet markets in Thailand [78]. *hilA* and *spi4R* differed by location type in the current study which may be due to outside influences and sources such as processing, transport and environmental conditions involved in abattoirs and wet markets. A meta-analysis involving diverse animal sources, geographical locations, and *Salmonella* revealed extensive serovar prevalence variations across different food animals such as pork, poultry, beef, and seafood but also geographical locations such as America, Asia, Africa, and Europe [79]. Similarly, Simpson et al. [80] showed variations in *Salmonella* serovar diversity indices in Australia, dependent on the sample type and environment (i.e., from humans to different domestic or wild animals). Moreover, natural or retail environments are also associated with serovars, such as Paratyphi B Java in seafood, natural environment, and wild mammals. These studies imply the indirect contribution of animal source, location, and other external variables to *Salmonella* serovar and subsequent virulence gene diversity as well as pathogenic potential.

This study has also shown that virulence gene frequencies can be related to serogroup variations. Although these frequencies may also be affected by differences in sample size, serogroup, or serovar genotypic virulence variations, previously documented. For instance, *Salmonella *Enteritidis within serogroup D (O:9) isolated from diarrheic children in a city of China also showed lower frequencies for *hilA* (83%) than in other serogroups (84–100%) but also showed a lower prevalence for *mgtC* (66%) than other serogroups (80–100%) [81]. Similarly, Thung et al. [54] showed serovar differences in frequencies of virulence genes *hilA*, *sopB*, and *stn* with a particularly drastic difference for *sopB,* which was not present in serovars London and Stanley. Hybridization techniques have also demonstrated distinguishing virulence gene patterns affecting their host range across different *Salmonella* serovars such as Typhimurium and Choleraesuis, and subsequent mutations of these regions contributed to decreased pathogenicity in vivo [82]. Furthermore, differences in virulence gene distributions among *Salmonella* serogroups may subsequently present varying degrees of pathogenicity. In a study by Rakov et al. [83], functional associations and allelic variations of virulence factors using protein sequences including *sseC*, *avrA*, *pipB* were revealed among intestinal (noninvasive) and invasive *Salmonella* serovars. The study reported that proteins, such as SiiE (SPI4) were only present in the former while others, such as MgtB (SPI3) and SseL (SPI2) were only in the latter. Similarly, *Salmonella* characterization from sand lizards showed variations in virulence gene prevalence among rare serovars consequently affecting pathogenicity; serovar Telhashomer, which had no SPI1 genes, also showed the lowest adhesion and apoptosis induction in vitro [84]. Comparisons between a clinical *Salmonella* isolate, and two other serovars of different pathogenic potential showed higher disease severity in pigs based on fecal and histopathological scores among the clinical isolate and serovar Typhimurium than Derby, which was reflected accordingly in the absence of some virulence factors in Derby such as *lpf*, *stc*, *stj*, and *sodC1* [85]. These studies corroborate with current results in that *Salmonella* serogroups and serovars contain diverse and complex virulence determinants, which explain their variations in pathogenesis programs and subsequent clinical outcomes.

This study mainly focused on *Salmonella* SPIs 1-5 due to their wide distribution and documented contributions to pathogenicity and two plasmid-borne virulence genes, their associations, and statistical analyses with external factors and *Salmonella* serogroups. Some isolates (*n* = 212) were not serogrouped in previous studies, however, this does not affect the validity of virulence gene profiles of all 799 isolates or the serogroup and virulence gene predictions of 587 isolates. In addition, the potential limitations of this study are other SPI or non-SPI virulence genes, and serovar-level analysis which can provide more in-depth genotypic characterization of *Salmonella* and their pathogenic insights. The associations and predictions in this study may thus be underestimated due to these caveats as there are extensive diversities in *Salmonella* serovars and virulence genes.

## Conclusions

The high prevalence and co-occurrence of virulence genes *mgtC*, *pipB*, *avrA*, *hilA*, *spi4R*, and *sseC* support the wide distribution of SPIs 1-5 across *Salmonella*, their high pathogenic potential which present food safety and public health concerns and provide a wealth of virulence data of *Salmonella* in the Philippines. Statistical analyses also determined the predictability of virulence genes namely, *hilA, sseC*, *spi4R*, and *pipB* based on animal sources and location types, which suggests the contributions of external factors to *Salmonella* strains present and their subsequent pathogenicity. *Salmonella* serogroups have also been shown in this study to predict the presence of virulence genes *avrA*, *hilA*, *sseC*, and *mgtC*, which suggest the diversity of virulence gene distributions across *Salmonella* and thus emphasizes the complexity of their pathogenesis program. Hence, further studies to elucidate the complex mechanisms of SPI crosstalk and associations of virulence determinants across different *Salmonella* serovars are needed to define what facilitates the extensive clinical manifestations of *Salmonella* infections. While this study detected SPIs 1-5 virulence genes, future studies on the prevalence of virulence genes from other SPIs are recommended. In addition, more studies on abattoirs, wet markets and particularly farms, different food animal sources and *Salmonella* serovars to further elucidate contributions of external and internal factors to *Salmonella* virulence are recommended. Associations between virulence and antimicrobial resistance can also be explored among pathogens not limited to *Salmonella*. The authors also recommend policymakers in the Philippines to reinforce and re-evaluate guidelines and regulations within food animal industries involving the entire chain and expand surveillance and monitoring to protect farmers, retailers, and consumers alike.

## Data Availability

The datasets used and/or analyzed during the current study are available from the corresponding author upon reasonable request.

## Abbreviations

SPI: *Salmonella* pathogenicity island
PCR: Polymerase chain reaction
T3SS: Type III secretion system
T1SS: Type I secretion system
TSB: Trypticase soy broth
XLD: Xylose lysine deoxycholate agar
NA: Nutrient agar
TE: Tris-EDTA buffer
ATCC: American Type Culture Collection
TAE: Tris-Acetate-EDTA buffer
